# It's All in How You Think About It: Construal Level and the Iowa Gambling Task

**DOI:** 10.3389/fnins.2016.00002

**Published:** 2016-01-22

**Authors:** Bradley M. Okdie, Melissa T. Buelow, Kurstie Bevelhymer-Rangel

**Affiliations:** Psychology, The Ohio State University at NewarkNewark, OH, USA

**Keywords:** decision making, construal level theory, Iowa Gambling Task, learning

## Abstract

Recent research has identified a number of factors that can influence performance on the Iowa Gambling Task (IGT) when it is used in clinical or research settings. The current studies examine the effects of construal level theory (CLT) on the IGT. Study 1 suggests that when primed with a high construal mindset (i.e., thinking abstractly vs. concretely), individuals learned to avoid Deck A more than those primed with a low construal mindset. Study 2 suggests that when construal level is manipulated through psychological distance (i.e., selecting for a close vs. distant friend), individuals in a high construal mindset instead showed a preference for Deck A compared to individuals in a low construal mindset or a control group. Taken together, these studies suggest that IGT performance is impacted by the manner in which one construes the task. Implications for decision making research and use of the IGT as a clinical and research instrument are discussed.

## Introduction

People make decisions daily, from seemingly mundane choices like what to wear to major life decisions like who to marry or what career path to take. Whether mundane or life-changing, the decisions we make define who we were, who we are, and who we will be. Individuals who choose advantageously reap the benefits of those decisions, while those that choose disadvantageously are often left wondering how they arrived at their current state. Many measures exist that assess different types of decision making, some of which also purport to predict who will decide advantageously and who will decide disadvantageously. The Iowa Gambling Task (IGT; Bechara et al., 1994) is widely used by clinicians and researchers alike, and examines both advantageous and disadvantageous selections under ambiguity and risk.

Recent research has called into question the utility of using the IGT in isolation as a clinical measure of decision making, as significant fluctuations in performance occur in a healthy control populations and the precise decision making measured by the task is still debated (for review, see Steingroever et al., 2013). Prior research has highlighted how contextual factors such as negative mood and extra learning trials can improve performance on the IGT (Buelow et al., 2013, 2014), while the anticipation of a public speaking task can decrease performance on the IGT (Preston et al., 2007) suggesting that contextual factors could mask or otherwise interfere with the assessment of the individual's actual (baseline) decision making ability. Additionally, the IGT scoring criteria can affect interpretation of selections on the task (see below for additional detail). Taken together, these contextual and scoring factors can affect performance on behavioral measures of decision making. Moreover, multiple studies have shown few significant correlations between performance on the IGT and performance on other behavioral decision making and problem solving tasks (Lejuez et al., 2003; Overman et al., 2004; Aklin et al., 2005; Skeel et al., 2007; Buelow and Blaine, 2015), leading to questions regarding whether the task is sensitive to only decision making impairments. The IGT (Bechara, 2007) is used by clinicians to assess risk-taking behavior and decision making. Understanding the different contextual factors that could influence performance on this task will help guide the improvement of the clinical assessment of decision making. Given this, the present set of studies sought to examine whether the manner in which the task is construed (abstractly or concretely) might affect outcomes on the IGT.

## The iowa gambling task as a clinical measure of decision making

The IGT is one of the most widely cited behavioral decision making tasks in the literature and has been adapted into a clinical assessment instrument (Bechara, 2007) based on the Bechara et al. (2001) test revision. The task was initially designed to assess decision making impairments in individuals with ventromedial prefrontal cortex damage who showed real-world decision making failures but scored within the normal range on formal measures of executive functioning (Bechara et al., 1994). Although originally designed for individuals with focal lesions, research has shown the IGT is sensitive to impairments due to head injury, amygdala damage, bipolar disorder, obsessive compulsive disorder, pathological gambling, substance abuse and dependence, and attention-deficit/hyperactivity disorder (see Buelow and Suhr, 2009, for discussion). Typically, poor performance on the IGT (i.e., choosing disadvantageously) is associated with the presence of these and other neurological or psychological diagnoses.

On the IGT, participants are given a loan of $2000 and told to maximize profit over 100 trials by selecting from one of four decks of cards: A, B, C, or D. On each trial, participants always experience a win but sometimes also experience a loss. With some decks, those losses can outweigh the benefits of the immediate reward. Decks A and B have an average profit of $100 per selection and Decks C and D have an average profit of $50 per selection (Bechara, 2007). After 10 selections from Decks A or B, individuals have incurred a net loss of $250; however, after 10 selections from Decks C or D, individuals instead have earned a net gain of $250 (Bechara, 2007). From these differences in long-term outcomes, Decks A and B have been considered “disadvantageous” and Decks C and D “advantageous” (Bechara, 2007). Differences exist between Decks A and B and Decks C and D, based on the frequency of losses. Selections from Decks A and C experience losses on 50% of trials, whereas selections from Decks B and D experience losses on only 10% of trials (Bechara, 2007). These differences in frequency of losses may explain why a significant subset of “healthy control” participants exhibit a preference for Deck B, with high immediate rewards, a low frequency of losses, but long-term negative outcomes (Toplak et al., 2005; Caroselli et al., 2006; Fernie and Tunney, 2006; Buelow et al., 2013).

The IGT creators put forth that the task was sensitive to affective decision making (i.e., gut feelings and intuition; Bechara et al., 1994; Damasio, 1994; Seguin et al., 2007). Research supports this idea, indicating that individuals develop somatic markers in response to disadvantageous selections on the task that help guide performance (Bechara et al., 1996, 1997; Crone et al., 2004). However, recent research on the somatic marker hypothesis is mixed (Dunn et al., 2006), with some research suggesting that the IGT assesses both affective and deliberative decision making at different points in the task (Maia and McClelland, 2004; Brand et al., 2007; Guillaume et al., 2009; Schiebener et al., 2011). Although the precise decision making processes involved on the task are still being understood, most researchers agree early trials assess affective decision making while later trials assess deliberative decision making (Dunn et al., 2006; Wood and Bechara, 2014; Buelow and Blaine, 2015). To help further differentiate these two sets of trials, Brand et al. (2007) found that decision making during the early trials (“decision making under ambiguity”) is guided by gut feelings and instincts, while during the later trials (“decision making under risk”) participants have learned enough about the decks to estimate the relative risks and benefits of each. Supporting this distinction is the failure of the IGT to consistently correlate with other affective decision making tasks such as the Columbia Card Task-hot (CCT-hot; Figner and Voelki, 2004) and the Balloon Analog Risk Task (BART; Lejuez et al., 2002), suggesting that it measures a unique type of decision making not assessed in other decision making tasks. When factor analyzed, the IGT held as a unique factor in a model with the BART and CCT (Buelow and Blaine, 2015).

Despite the IGT's wide use in research and clinical practice, recent research has called into question its use as a stand-alone tool for investigating clinical decision making. Some have argued that the IGT can be influenced by different factors, including age (Blair et al., 2001; Crone and van der Molen, 2004; Kerr and Zelazo, 2004; Denburg et al., 2006; Fein et al., 2007; Garon and Moore, 2007), gender (Reavis and Overman, 2001; Bolla et al., 2005; Davis et al., 2007; Goudriaan et al., 2007; Businelle et al., 2008; van den Bos et al., 2013), personality (Addison and Schmidt, 1999; van Honk et al., 2002; Crone et al., 2003; Franken and Muris, 2005; Suhr and Tsanadis, 2007; Buelow and Suhr, 2013), extra learning trials (Buelow et al., 2013, 2014; Lin et al., 2013), and mood (Must et al., 2006; Suhr and Tsanadis, 2007; Buelow et al., 2013). It is important to acknowledge that contextual factors likely affect many clinical measures. However, the effects of contextual factors become paramount when a lack of agreement exists on what a specific instrument, such as the IGT, is truly measuring. Understanding what and how contextual factors affect performance is an important way to gain knowledge of the test's ability to measure what it was designed to measure. To fully understand an individual's decision making processes, assessment of the construct should be minimally sensitive to contextual factors. Although some of these factors may be more consistent across time (i.e., gender, personality) than others (i.e., age, mood), some factors (i.e., extra trials) are products of the testing situation. It is possible that the individual's mindset—not just current mood—may affect testing performance on the IGT. Despite these known limitations of the IGT and inconsistencies in how it is scored, it has been put forth as a clinical assessment instrument; however, no other behavioral decision making tasks have been adapted for clinical use alongside the IGT. It is important, then, to understand the different factors that influence performance on this task.

## IGT and construal level

As previously stated, the IGT can be broken down into disadvantageous (Decks A and B) and advantageous (Decks C and D) deck choices (Bechara et al., 1994), with advantageous decision making dependent on consideration of long-term rather than short-term outcomes (as the authors originally intended). However, this masks differences in the frequency of wins and losses between Decks A and B and between Decks C and D. Although Decks A and B have similar long-term outcomes, selecting from Deck A results in losses on 50% of trials whereas Deck B experiences losses on only 10% of trials (Bechara, 2007). The magnitude of losses is therefore greater with Deck B than Deck A. A similar pattern emerges for Decks C (50% losses) and D (10% losses, greater magnitude of losses). Thus, when selections from each deck are analyzed independently, the IGT can also be conceptualized as a choice between high and low frequency of wins and losses (Caroselli et al., 2006; Lin et al., 2007, 2009; Chiu et al., 2008). Due to these differing foci of attention (long-term outcomes vs. frequency of losses), the IGT manual now refers to selections from Deck B as a non-optimal decision making strategy but continued selection from Deck A as indicative of pathological decision making (Bechara, 2007). Not examining the individual deck selections separately may result in someone who chooses from Deck B, to minimize frequency of losses, labeled as just as disadvantageous a decision maker as someone who continually selects from Deck A despite the negative outcomes. One contextual factor that has not been investigated with the IGT, but that may affect outcomes on individual deck selections, is the manner in which individuals construe the task.

Individuals often imagine future situations in both the near and distant future. For example, an individual may imagine what their life might be like next week or in 5 years. Considerable research suggests that the process by which an individual mentally imagines near and distant future events differs leading to variable outcomes in such domains as category breadth (Liberman et al., 2002, study 1), dimensionality of future representations (Liberman et al., 2002, study 2), and optimism (Taylor and Brown, 1988). Construal level theory (CLT; Trope and Liberman, 2003, 2010) suggests that when individuals imagine future events, they create abstract mental representations that vary to the extent that the imagined future is near or distant. Thus, CLT suggests that individuals can construe future events abstractly (high level construal) or concretely (low level construal). Research indicates that individuals will construe near future events using a low level construal and distant future events using a higher-level construal (Trope and Liberman, 2010). Moreover, CLT suggests that the use of a high level construal increases with psychological distance (Semin and Fiedler, 1991; Fujita et al., 2006a). Construal level differences have also been shown using other types of psychological distance, such as temporal and social distance (Liberman et al., 2007). For example, Fujita et al. (2006a) had participants watch a video of two students interacting and were informed the students were physically near or physically distant from the participant. Participants then provided a written description of the activity in the video. Results indicated descriptions for those in the physically distant condition contained more abstract language compared to those in the physically near condition, suggesting that participants construed the activity in the video with greater abstraction when they believed the location was distal rather than proximal.

Applied to the IGT, imagining that one is earning money for a distant acquaintance (high level construal) should lead to advantageous decision making while imagining earning money for a close friend (low level construal) should lead to disadvantageous decision making. Although the task directions do not indicate the individual should imagine earning money for a close friend, it is possible that recent knowledge of a friend's financial hardships could weigh on the individual's mind, in turn affecting performance on the IGT. Alternatively, if one actively engaged in abstract thought prior to taking the IGT, that construal process could transfer to the IGT affecting outcomes (see Smith and Branscombe, 1988; Gollwitzer and Kinney, 1989; Förster et al., 2004; for examples using non-IGT tasks).

CLT also posits that when individuals are in a high construal mindset they are more likely to rely on their internal values compared to when they are in a low construal mindset (Sagristano et al., unpublished manuscript). For example, when students are informed that a potential class is temporally distant (e.g., will take place in 1 year) they are more likely to focus on whether or not the professor treats students with respect. However, when students believe that a potential class is temporally close (e.g., will start in few days) they are more likely to focus on things such as the professor's typical grade distribution (Kivetz and Tyler, 2007; Torelli and Kaikati, 2009). Moreover, individuals are more likely to endorse attitude consistent behavior when they are imagining the behaviors in the distant rather than near future (Fujita et al., 2006b; Sagristano et al., unpublished manuscript). Thus, decisions made in the future are more likely to be guided by and reflect one's internal values and desires while decisions made in the present are more likely to reflect specific features of the decision (e.g., contextual and situational factors).

Given the extent to which different construal mindsets can affect the processing of information, it stands to reason that these psychological states may affect performance on decision making tasks in which differences in long-term vs. short-term outcomes are present, such as the IGT (e.g., Buelow et al., 2013), the Delay Discounting Task (e.g., Kirby and Herrnstein, 1995; Bickel et al., 1999; Kirby et al., 1999, 2005), and the BART (e.g., Acheson et al., 2007; Benjamin and Robbins, 2007; Lejuez et al., 2002). That is, individuals in a high construal mindset should be more likely to act in accordance with their internal preferences and values, leading to more advantageous choices on decision making tasks when the goal of the task is congruent with a focus on long-term outcomes (e.g., winning more money on the IGT). More importantly, those in a high construal state of mind are more likely to focus on abstractions (rather than specifics) leading to more advantageous decision making on the IGT and other tasks in which short- and long-term outcomes are available. That is, individuals in a high construal mindset are likely to ignore concrete and specific aspects of the decision making task and focus instead on core and stable elements of the task. In relation to the IGT, individuals in a high construal mindset may ignore the immediate consequence of their actions (e.g., large win vs. large loss) and instead focus on the more abstract goal-congruent long-term consequences (e.g., earn more money). One could also consider that decision making on tasks such as the IGT requires that one parse several pieces of information to learn to choose advantageously on the task. During the IGT, individuals are presented with win vs. loss information, but also large vs. small immediate outcomes. The ability of individuals to choose advantageously on the task requires that they ignore some information (large immediate rewards that lead to less money overall—favorable short-term outcomes) in favor of other information (small immediate rewards that lead to more money overall—favorable long-term outcomes). The ability to ignore irrelevant information to engage in goal-directed behavior should be enhanced when one is in a high (vs. low) construal mindset. Previous research has suggested that individuals in a high construal mindset focus more on attaining goals, whereas individuals in a low construal mindset focus more on avoiding losses (Pennington and Roese, 2003; Förster and Higgins, 2005; Lee et al., 2010). In addition, high construal level is associated with a focus on the “pros” or the positives of a given decision (Eyal et al., 2004), indicating participants in this mindset may begin to show a preference for the advantageous decks as information is learned and the task progresses. In a recent study, Lermer et al. (2015) found greater risk-taking on the BART among individuals in a high-construal mindset; however, no learning of risks and benefits associated with decisions occurs on the BART compared to the IGT.

## The present studies

The present studies examined whether the manner in which you construe the task affects performance on the IGT. Previous research has shown that some contextual variables can affect performance, and it is possible that the mindset one is in during testing—high vs. low construal—can also affect performance. We believe that construal mindset serves as an attribute of the decision making process. Understanding all of the mechanisms negatively affecting IGT performance is important as the IGT is frequently used by researchers and clinicians alike. Across two studies, participants were primed with a high- or low-level construal procedural task, or received no prime, and then completed the IGT. We predicted that those primed with a high construal mindset would choose more advantageously on the IGT compared to those primed with a low level construal mindset.

## Study 1 method

### Participants

Participants were 90 undergraduate students (58 females) at a regional campus of a large Midwestern university, ages 18–33 (*M* = 19.00, *SD* = 2.30), who were enrolled in General Psychology courses. Most (67.4%) self-identified as Caucasian.

### Measures

#### Iowa gambling task (IGT)

In the present study, the IGT version available through PAR, Inc. was utilized (Bechara, 2007). This version of the IGT is based on the revised task described in Bechara et al. (2001). The task has been validated in various patient and non-patient samples (see Buelow and Suhr, 2009, for a review). Based on the scoring issues outlined previously and the confounding of long-term outcomes and frequency of losses on the decks, the percent selections from each individual deck (A,B,C,D) during decision making under ambiguity (Trials 1–40) and decision making under risk (Trials 41–100; Brand et al., 2007) were calculated.

### Procedure

The present study was approved by the University's Institutional Review Board. Following informed consent, participants were randomly assigned to complete a construal level manipulation utilizing procedural mindset priming—the categories vs. exemplars task (Fujita et al., 2006b). In this task, participants are provided with 40 stimulus words and asked to repeatedly engage in high- or low-level construal processing by generating a superordinate category label (high construal; *n* = 30 participants) or subordinate exemplar (low construal; *n* = 30 participants) for each such that the induced mindset transfers to a subsequent task (see Smith and Branscombe, 1988; Gollwitzer and Kinney, 1989; Förster et al., 2004; for examples). For example, those induced into a low construal mindset might be given the word “soda” and provide “Coke” as their response, while those induced to a high construal mindset may provide “beverage” as their response. Immediately following this manipulation, participants completed the IGT.

An additional 30 participants made up a control group who did not complete a construal manipulation. These participants were compiled from a separate study conducted concurrently to the present study, in which participants did not receive a manipulation prior to the IGT (Bevelhymer-Rangel and Buelow, 2014).

### Data analysis

Data were first examined for between-groups differences in demographic variables. Repeated-measures ANOVAs were conducted on the IGT deck selections, with study condition (high construal, low construal, control) as the between-subjects variable and block (Block 1: Trials 1–40; Block 2: Trials 41–100) as the repeated-measures variable.

## Study 1 results and conclusions

There was not a significant difference between the three groups in terms of age, *F*_(2, 86)_ = 2.67, *p* = 0.08; or gender, $\chi_{\left(2,\;N=90\right)}^{2}=3.01$, *p* = 0.22. For Deck A, there was a significant main effect of block, *F*_(1, 87)_ = 47.96, *p* < 0.001, in that participants selected more from Deck A in Block 1 than Block 2 (see Table 1 for means, standard deviations, and effect sizes). Thus, learning occurred on the IGT, as participants shifted their decisions away from the most disadvantageous deck. There was also a significant main effect of group, *F*_(2, 87)_ = 3.63, *p* = 0.03. Participants in the Low Construal group selected significantly more from Deck A than participants in the High Construal group, *p* = 0.01. In addition, the control group selected marginally more from Deck A than participants in the High Construal group, *p* = 0.067. The interaction was not significant for Deck A, *F*_(2, 87)_ = 0.13, *p* = 0.88.

**Table 1:** **Study 1 Variables Presented as Mean (standard deviation)**.

**Variable**	**Block 1**	**Block 2**	**η^2^ B**	**η^2^ G**	**η^2^ B × G**
**Deck A**			0.355	0.077	0.003
High	18.33 (6.27)	12.06 (6.23)			
Low	21.50 (5.82)	15.67 (8.44)			
Control	21.08 (5.90)	14.11 (6.83)			
**Deck B**			0.009	0.022	0.001
High	36.00 (10.96)	34.61 (20.92)			
Low	33.75 (8.45)	31.50 (14.53)			
Control	32.00 (8.47)	30.56 (18.05)			
**Deck C**			0.041	0.001	0.004
High	21.42 (6.84)	22.33 (11.22)			
Low	20.83 (5.74)	23.61 (13.63)			
Control	21.92 (6.78)	23.05 (15.37)			
**Deck D**			0.123	0.001	0.001
High	24.25 (10.69)	31.00 (18.30)			
Low	23.92 (8.03)	29.72 (13.35)			
Control	24.92 (8.89)	30.48 (17.29)			

B, Block; G, Group; B × G, Block × Group Interaction; Deck, percent selections from Decks A, B, C, and D on Block 1 (Trials 1–40) and Block 2 (Trials 41–100).

For Deck B, none of the main [Block: *F*_(1, 87)_ = 0.82, *p* = 0.37; Group: *F*_(2, 87)_ = 0.99, *p* = 0.38] or interaction, *F*_(2, 87)_ = 0.02, *p* = 0.98, effects were significant. Similarly, none of the main [Block: *F*_(1, 87)_ = 1.21, *p* = 0.28; Group: *F*_(2, 87)_ = 0.04, *p* = 0.96] or interaction, *F*_(2, 87)_ = 0.16, *p* = 0.85, effects were significant for Deck C.

Finally, for Deck D, the main effect of Block was significant, *F*_(1, 87)_ = 12.24, *p* = 0.001. Participants selected significantly more from Deck D during Block 2 than Block 1, *p* = 0.001. Neither the main effect of group, *F*_(2, 87)_ = 0.06, *p* = 0.94, or the interaction effect, *F*_(2, 87)_ = 0.04, *p* = 0.96, were significant.

Taken together, the present results indicate no significant interaction between group and IGT block; however, significant main effects emerged on Decks A and D. Specifically, learning occurred on the task: participants, independent of group, learned to avoid Deck A and select from Deck D as the task progressed. These results are consistent with previous research showing the IGT does not solely rely on affective decision making processes, and instead learning can occur (Maia and McClelland, 2004; Dunn et al., 2006; Brand et al., 2007; Guillaume et al., 2009; Schiebener et al., 2011; Wood and Bechara, 2014; Buelow and Blaine, 2015). A significant main effect of construal level group emerged on Deck A, in that participants primed with a low construal mindset selected significantly more from Deck A (independent of block) than participants in a high construal mindset. In addition, the high construal group selected marginally less from Deck A than the control group. The results of Study 1 provide initial evidence that a high-level construal mindset can lead to a more advantageous decision making strategy on the IGT, in that participants learned to avoid Deck A—a deck associated with more pathological decision making (Bechara, 2007). However, to ensure that a high-level construal mindset led to changes in decision making strategy, we decided to replicate Study 1 using a different type of construal manipulation in which participants were asked to earn money on the IGT for a mere acquaintance (high-level construal) or a close friend (low-level construal). In a recent study, Kim et al. (2013) found that the greater the perceived psychological distance from a person, the more advantageous decisions were made. In the present study, it was hypothesized that participants in the high construal condition would again select more advantageously than those in the low construal condition.

## Study 2 method

### Participants

Participants were 90 undergraduate students (44 females) at a regional campus of a large Midwestern University who were 18–34 years old (*M* = 18.92, *SD* = 1.99) and were enrolled in General Psychology courses. Most (72.4%) self-identified as Caucasian.

### Measures

#### Iowa gambling task (IGT)

The standard computerized IGT was again utilized (Bechara, 2007). The percent selections from each individual deck during early (Trials 1–40) and later (Trials 41–100) trials were calculated.

### Procedure

The present study was approved by the University's Institutional Review Board. Following informed consent, participants were randomly assigned to complete a different construal level manipulation than in Study 2. This construal manipulation involved manipulating psychological distance rather than procedural mindset (Kim et al., 2013). Participants were either asked to think of someone very close to them—a close friend or family member (low construal; *n* = 30 participants)—or to think of someone not very close to them—a mere acquaintance (high construal; *n* = 30 participants). Participants in both groups were then asked to make decisions on the IGT as if they were earning money for that individual instead of themselves.

An additional 30 participants made up a control group who did not complete a construal manipulation. These participants were compiled from a separate study conducted concurrently to the present study, in which participants did not receive a manipulation prior to the IGT, and were distinct from the control group utilized in Study 1 (Bevelhymer-Rangel and Buelow, 2014).

### Data analysis

Data were first examined for between-groups differences in demographic variables. Repeated-measures ANOVAs were conducted on the IGT deck selections, with study condition (high construal, low construal, control) as the between-subjects variable and block (Block 1: Trials 1–40; Block 2: Trials 41–100) as the repeated-measures variable.

## Study 2 results and conclusions

There was not a difference between groups in terms of gender, $\chi_{\left(2,\;N=89\right)}^{2}=5.05$, *p* = 0.08. There was, however, a significant difference in age, *F*_(2, 85)_ = 3.33, *p* = 0.04. The control group (*M* = 18.18, *SD* = 0.48) was significantly younger than the high construal group (*M* = 19.47, *SD* = 2.93), *p* = 0.04. Due to this age difference, correlations were calculated between age and IGT performance. Age was significantly correlated with Block 1 selections for Deck C, *r*_(86)_ = 0.26, *p* = 0.02, and Deck D, *r*_(86)_ = −0.23, *p* = 0.03, only. Given this minimal relationship between age and performance on the IGT and our small sample size, we elected not to include age as a covariate in the remaining analyses.

For Deck A, there was a significant main effect of Block, *F*_(1, 87)_ = 36.15, *p* < 0.001, in that participants selected more from Deck A in Block 1 than Block 2, *p* < 0.001, independent of group (see Table 2 for means, standard deviations, and effect sizes). The group effect was marginal, *F*_(2, 87)_ = 2.91, *p* = 0.06, in that participants in the high construal group selected more from Deck A than participants in the control (*p* = 0.025) or low construal (*p* = 0.073) groups. There was also a significant block by group interaction, *F*_(2, 87)_ = 6.94, *p* = 0.002. Participants in the control group selected significantly more from Deck A in Block 1 than Block 2, *p* < 0.001. In addition, participants in the low construal group selected significantly more from Deck A in Block 1 than Block 2, *p* < 0.001. There was no difference in selections across blocks for the high construal group, *p* = 0.59.

**Table 2:** **Study 2 variables presented as mean (standard deviation)**.

**Variable**	**Block 1**	**Block 2**	**η^2^ B**	**η^2^ G**	**η^2^ B × G**
**Deck A**			0.294	0.063	0.138
High	21.33 (8.25)	20.11 (14.00)			
Low	24.00 (9.25)	11.39 (7.00)			
Control	21.58 (5.51)	12.22 (6.73)			
**Deck B**			0.004	0.016	0.054
High	32.83 (16.88)	27.44 (13.6)			
Low	31.00 (10.48)	28.67 (18.45)			
Control	31.08 (9.46)	35.44 (19.06)			
**Deck C**			>0.001	0.017	0.028
High	23.25 (13.54)	22.22 (10.56)			
Low	22.00 (7.38)	24.94 (20.37)			
Control	21.50 (4.76)	19.06 (12.21)			
**Deck D**			0.166	0.012	0.011
High	22.58 (11.79)	30.28 (18.53)			
Low	23.00 (8.74)	35.00 (19.82)			
Control	25.83 (9.54)	33.28 (22.73)			

B, Block; G, Group; B × G, Block × Group Interaction; Deck, percent selections from Decks A, B, C, and D on Block 1 (Trials 1–40) and Block 2 (Trials 41–100).

For Deck B, neither of the main effects were significant [Block: *F*_(1, 87)_ = 0.38, *p* = 0.54; Group: *F*_(2, 87)_ = 0.70, *p* = 0.50]. In addition, the interaction effect was not significant, *F*_(2, 87)_ = 2.51, *p* = 0.09. For Deck C, again neither of the main effects were significant [Block: *F*_(1, 87)_ = 0.02, *p* = 0.90; Group: *F*_(2, 87)_ = 0.77, *p* = 0.47]. The block by group interaction was also not significant for Deck C, *F*_(2, 87)_ = 1.27, *p* = 0.29.

For Deck D, the main effect of block was significant, *F*_(1, 87)_ = 17.30, *p* < 0.001, in that participants selected more from Deck D in Block 2 than Block 1. The main effect of group was not significant, *F*_(2, 87)_ = 0.54, *p* = 0.58. The block by group interaction was also not significant, *F*_(2, 87)_ = 0.46, *p* = 0.63.

Counter to our hypothesis, individuals who were told to imagine earning money for a psychologically close other (low construal) learned to avoid Deck A on the IGT compared to those that imagined earning money for a psychologically distant other (high construal). Thus, participants in a high construal mindset selected from the riskiest deck, even more so than those who did not complete a construal priming task. Both the control group and the low construal group significantly decreased their selections from Deck A as the task progressed. In a series of three studies, Beisswanger et al. (2003) found that participants made riskier decisions for their friends than for themselves; however, this difference disappeared when the likelihood of significant negative outcomes increased. As applied to the IGT, it is possible that our participants felt that the potential for loss when making decisions for a close friend outweighed the potential for loss when making decisions for a distant friend, leading to improved decision making on the task.

## General discussion

While research is still being conducted on the task, clinicians are increasingly using the IGT to clinically evaluate decision making. Although the IGT manual (Bechara, 2007) indicates that the task can be used in this manner, recent research suggests that using the task as a sole determinant of decision making ability may not be an accurate measure of one's decision making ability (for review, see Steingroever et al., 2013). The purpose of this set of studies was to investigate whether the manner in which individuals construe the task affects IGT performance.

Across two studies, manipulating the manner in which individuals construed the task differentially affected outcomes on the IGT. When using procedural mindset priming to manipulate the manner in which individuals construed the IGT (Study 1), individuals in a high-level construal mindset learned to avoid Deck A, the deck associated with pathological decision making (Bechara, 2007). Conversely, when manipulating psychological distance to manipulate construal level (Study 2), individuals in a high construal mindset failed to learn to avoid Deck A. Instead, individuals in a low construal mindset and control participants learned to avoid Deck A as the task progressed. Thus, the manipulations produced results in opposition to one another, suggesting the two may affect scores on the IGT using separate psychological mechanisms.

There are several reasons why procedural mindset priming might operate differently on the IGT compared to a manipulation involving psychological distance. Both manipulations attempt to illicit an abstract or concrete mindset, but do so in fundamentally different ways. Procedural mindset priming hinges on the assumption that repeatedly engaging in a particular type of processing style (e.g., high construal) primes that mindset, making it more likely for that processing style to transfer to an immediately subsequent task. Like most priming effects, its effectiveness is likely short-lived and dependent upon the contiguity of the first and second tasks (see Van den Bussche et al., 2009, for discussion of priming effects). To this end, construal level procedural mindset priming effects should be strongest early in the subsequent task and should fade as that task progresses. It is possible that with administration of additional trials, which has been shown to improve performance on the IGT (Buelow et al., 2013; Lin et al., 2013), the effects of construal level priming may dissipate and exert less of an influence on task performance.

In both studies, significant effects emerged on Decks A and D only, which may be attributed to individual deck-level differences. Deck D is widely regarded as the “best” or most advantageous deck, as continued selections from it result in long-term gains and a lower frequency (10%) of losses (Bechara, 2007). Deck C, although considered an advantageous deck, produces losses on 50% of trials. Although in the long term the wins outweigh the losses from this deck, individuals who are attuned to loss frequency may avoid this deck and deem it a disadvantageous deck. Previous research has shown a prominent Deck B preference among healthy control participants (e.g., Toplak et al., 2005; Caroselli et al., 2006; Fernie and Tunney, 2006), and it appears that this effect is driven by a preference for high immediate gains (in comparison to the lower immediate gains of Deck D) and the low frequency of immediate losses. Thus, for a significant subset of healthy controls, Deck B can be seen as an advantageous deck. It is possible that our lack of findings with Decks B and C are due in part to these confounds in how the decks are appraised by participants. Deck A selections are indicative of pathological decision making (Bechara, 2007), and continued selections from this deck are not typically seen in control participants. It is then possible the outcomes for Deck A in the present studies are the result of faster detection of the disadvantages of Deck A due to the manner in which participants construed the task. It is also possible that characteristics of our sample led to these deck-specific results. College student participants may exhibit the prominent Deck B phenomenon, as has been shown in some of the previous research (e.g., Caroselli et al., 2006). In addition, students may have multiple friends or family members experiencing financial strain—or may be experiencing this strain themselves—thus leading to a greater emotional investment in decisions made for close friends in Study 2. We also had more females than males in Study 1, and gender may have played a role in deck selections (e.g., Bolla et al., 2005; Davis et al., 2007; Businelle et al., 2008).

Across two studies, we have provided evidence that construal level can impact performance on the IGT. Specifically, increasing psychological distance may lead to continued selections from a disadvantageous deck, while priming individuals with a high construal mindset may lead to decreased selections from this same disadvantageous deck. It is possible that during a clinical evaluation, individuals engaging in high construal level thinking (planning for their retirement) prior to taking the IGT may likely make more advantageous decisions than those engaging in low construal level thinking prompted by psychological distance (imagining a close other), or even compared to their own decisions when not engaging in high construal level thinking. Additionally, during a clinical evaluation with the IGT, a participant may be thinking about their own recent financial difficulties, or those of a close friend or family member. Each of these processes is likely to impact performance on the IGT and, more importantly, the clinical evaluation of decision making impairment. In addition, these same contextual factors can negatively affect assessment of decision making in a lab-based setting. The IGT is still frequently used as a behavioral measure of risky decision making, and without taking contextual factors such as construal level into account, ensuring understanding of what the IGT is assessing is difficult. However, it is important to note that construal level is not the only factor affecting IGT performance, as it is likely that other factors (such as age, gender, personality, and other to-date unknown factors) can also affect performance on this task in the clinic and in the lab.

The results between the present studies were contradictory. Thus, while these differences may be accounted for by the type of manipulation used, the results should be interpreted with caution until further research can identify the exact psychological mechanism responsible for those differences or appropriate moderators can be identified. Previous research has shown that emotional state can affect decision making (Forgas, 1995), with both positive (Nygren et al., 1996; Roiser et al., 2009) and negative (Heilman et al., 2010; Buelow et al., 2013) mood affecting performance on the IGT. Within the moral decision making field, personal dilemmas elicit greater emotional response than impersonal or non-moral dilemmas (Greene et al., 2001; Skoe et al., 2002; Myyry and Helkama, 2007), which in turn affects decision making. It is possible that in Study 2, participants who were asked to decide for a close friend increased their emotional involvement and engagement on the IGT. As the IGT is thought to be based, at least in part, on emotion-based decision making, it is possible that this activation of an emotional experience increased decision making whereas making decisions for a distant acquaintance could have resulted in limited emotional input during the task. Additionally, our data were culturally homogenous and cannot account for the impact that culture may have on our findings. Individuals from different cultural backgrounds may interpret the construal tasks differently, in turn changing their assumed effects on cognitive processes.

Taken together, our findings suggest that priming participants with a high or low construal mindset affects advantageous and disadvantageous decision making on the IGT. Across multiple studies, personality, mood, and other contextual factors have been shown to affect performance on the IGT, a task designed to assess real-world decision making impairments among individuals with damage to the prefrontal cortex. Disadvantageous decision making is not specific to such damage, nor to a specific neurological or psychological diagnosis. It is important for clinicians to account for the presence of such contextual and other factors before determining a patient's decision making ability when using the IGT in isolation. Using the IGT as part of a multi-faceted approach to assessing decision making that includes the use of multiple decision making tasks should help increase the validity of the assessment, as contextual effects on any one task should be minimized when results are congruent across measures. However, to truly conduct a valid assessment of decision making utilizing multiple measures, additional measures validated for use in clinical populations are needed. This multi-faceted approach would then allow for a broader, and more accurate, understanding of decision making that is less resistant to contextual and other factors. Researchers investigating what the IGT is truly measuring should also be cognizant of contextual factors as they may affect outcomes on the IGT, leading to null relationships between it and other decision making tasks. Ultimately, clinical research informs clinical practice. Thus, a thorough understanding of the task and the factors that affect performance from lab-based settings is warranted prior to using the IGT as a stand-alone measure of decision making in clinical evaluations.

### Conflict of interest statement

The authors declare that the research was conducted in the absence of any commercial or financial relationships that could be construed as a potential conflict of interest.
